# Impact of Structured Postoperative Surveillance on Survival in Patients with Resected Pancreatic Adenocarcinoma

**DOI:** 10.3390/cancers17091424

**Published:** 2025-04-24

**Authors:** Anne Jacobsen, Maarten Flessa, Anna-Lena Abels, Franziska Czubayko, Anke Mittelstädt, Christian Krautz, Georg F. Weber, Robert Grützmann, Maximilian Brunner

**Affiliations:** Department of General and Visceral Surgery, University Hospital Erlangen, Friedrich-Alexander-University (FAU), 91054 Erlangen, Germany; anne.jacobsen@uk-erlangen.de (A.J.); maarten.flessa@fau.de (M.F.); anna-lena.abels@uk-erlangen.de (A.-L.A.); franziska.czubayko@uk-erlangen.de (F.C.); anke.mittelstaedt@uk-erlangen.de (A.M.); christian.krautz@uk-erlangen.de (C.K.); georg.weber@uk-erlangen.de (G.F.W.); robert.gruetzmann@uk-erlangen.de (R.G.)

**Keywords:** pancreatic cancer, pancreatic ductal adenocarcinoma, recurrence, surveillance, follow-up

## Abstract

Background: This study aimed to investigate whether structured surveillance after resection of PDAC has an impact on survival. Methods: 206 patients who underwent PDAC resection at the University Hospital Erlangen between 2005 and 2020 and survived for at least 90 days postoperatively were included in this retrospective study. The impact of surveillance (structured vs. no structured surveillance) respectively of recurrence patterns (recurrence in follow-up vs. symptomatic recurrence) on overall and disease-free survival was investigated. Results: A total of 157 patients (76%) participated in a structured surveillance program after pancreatic resection. During a mean follow-up of 28.5 months, 137 patients (67%) developed recurrence. Patients with structured surveillance showed significantly better outcomes for overall survival after surgery (OSaS: 29.2 months with structured surveillance vs. 16.4 months without, *p* < 0.001), overall survival after recurrence (OSaR: 10.8 months vs. 3.6 months, *p* < 0.001), and disease-free survival (DFS: 14.8 months vs. 11.4 months, *p* = 0.010). In a subgroup analysis of 112 patients with recurrence, survival benefits were observed for those whose recurrence was detected during follow-up compared to those with symptomatic recurrence (OSaS: 24.8 months in the follow-up group vs. 17.2 months in the symptomatic group, *p* < 0.001; OSaR: 12.6 months vs. 6.5 months, *p* < 0.001). Conclusion: This study provides evidence that structured surveillance after PDAC resection is beneficial for both overall and disease-free survival. However, randomized studies are needed to confirm the positive impact of structured surveillance programs on survival after pancreatic resection.

## Simple Summary

The present retrospective study, analyzing 206 patients with pancreatic ductal adenocarcinoma (PDAC) who underwent resection between 2005 and 2020 at the University Hospital Erlangen, provides further evidence that structured surveillance following pancreatic cancer surgery is associated with improved overall and disease-free survival. Patients who participated in a structured surveillance program had significantly better outcomes than those without structured follow-up. Additionally, patients whose recurrence was detected during routine follow-up had better survival rates compared to those with symptomatic recurrence. These findings highlight the potential benefits of structured postoperative surveillance, though further randomized studies are necessary to confirm the positive impact on survival outcomes.

## 1. Introduction

Pancreatic cancer is associated with a high mortality rate and an increasing incidence [1]. Although new multimodal therapy approaches, improved surgical techniques, as well as more effective chemo- and radiochemotherapies, have led to increasing survival rates, the poor prognosis, with a 5-year survival of around 10%, remains an unsolved problem [1,2,3].

Complete surgical resection of pancreatic ductal adenocarcinoma (PDAC) is known to be the only potential curative treatment. However, only 20–30% of all patients present with primary resectable tumor at diagnosis, and an additional 5–10% achieve secondary resectability after neoadjuvant therapy for borderline or locally advanced tumors. This highlights the critical importance of early detection of pancreatic carcinoma as a key factor in improving outcomes for this disease [4].

After PDAC resection, 5-year-survival rates of up to 30–40% are possible. However, even after resection, up to 80% experience cancer recurrence, mostly in the first two years after resection, preventing an even better prognosis [5,6,7,8,9,10]. Therefore, early detection of cancer recurrences could be a key factor in improving the survival of resected patients. Even though the treatment options for recurrent pancreatic cancer are limited at the moment, the continuously improving chemotherapy regimens may offer superior treatment for those patients in the future. However, to date, there is limited evidence for a structured postoperative surveillance in general, as well as regarding the optimal frequency and scale of postoperative surveillance after PDAC resection. Consequently, the consensus recommendations in the guidelines differ from symptom-focused surveillance to regular 3-monthly examinations, including imaging and blood tests, all providing only low levels of evidence [11,12,13,14,15].

A systematic review and meta-analysis by Halle-Smith et al. in 2021 indicated that structured surveillance after PDAC resection is more likely to detect cancer recurrence at an asymptomatic stage, thereby leading to earlier therapy and longer survival [16]. In a retrospective analysis of 125 PDAC patients, Zhang et al. also found a survival benefit for those whose cancer recurrence was detected during a scheduled follow-up [17]. However, the impact of structured surveillance programs after resection PDAC remains unclear due to a lack of high-level evidence [18]. Therefore, in the current German S3-guideline for pancreatic cancer, a structured follow-up is still not recommended [15].

The aim of this study was to evaluate the impact of structured surveillance after PDAC resection on the overall and disease-free survival of patients with primarily or secondary resectable PDAC.

## 2. Materials and Methods

**Figure 1:**
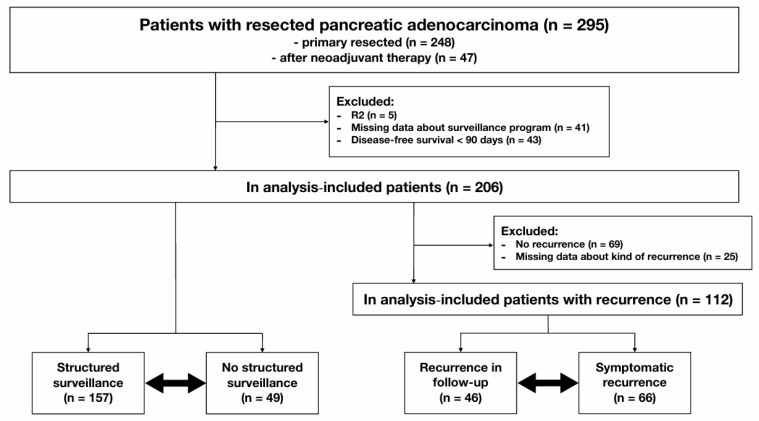
Flow chart of the study.

Prospectively collected data from the Erlangen Cancer Registry of the Department of Surgery were used for this retrospective analysis. We included all adult patients with PDAC who had pancreatic resection between 1 January 2005 and 31 December 2020 at the Surgical Department of the University Hospital Erlangen. Both primarily resectable and secondary resectable patients after neoadjuvant therapy were included. All patient cases were discussed in an interdisciplinary tumor board. Patients with primary resectable tumors were recommended for primary resection. Patients with pancreatic malignancies classified as borderline resectable or locally advanced were recommended for neoadjuvant therapy, followed by re-staging and potentially resection. Exclusion criteria were postoperative R2 status, disease-free survival shorter than 90 days—as these patients could not have structured surveillance—and missing data about postoperative surveillance (Figure 1).

Patients’ clinical data were collected from the clinical information system. Pathological and survival data for all patients were obtained from the above-mentioned Erlangen Cancer Registry. For histopathological description, the TNM-Classification of malignant tumors by the Union for International Cancer Control (UICC) (according to the 8th edition from 2017) was used [19]. Morbidity was assessed by the Clavien–Dindo classification [20].

Data on the surveillance and recurrence status of the mentioned patients were retrieved from the clinical information system. To gather information about patients who underwent postoperative surveillance outside the University Hospital Erlangen, we contacted the patients, their families, and their general practitioners by phone and mail. Based on the collected information about the surveillance and recurrence status, all included patients were grouped into two categories: those with structured surveillance and those without structured surveillance. Additionally, all patients with recurrence were categorized into two groups: those with recurrence detected during surveillance and those with recurrence detected by symptoms (Figure 1).

The retrospective study was approved by the local ethics committee (22-165-Br).

### 2.1. Definition of Structured Surveillance

Our current in-house clinical standard for structured surveillance includes CT scans of the thorax and abdomen, as well as the tumor markers CEA and CA 19-9 every three months during the first two years, every six months in years three and four, and once annually in year five. A PET-CT was not routinely used as part of the surveillance protocol.

However, for the purpose of the study, structured surveillance was pragmatically defined as at least one structured surveillance examination per year, including assessment of the tumor markers CEA and CA 19-9 and imaging via ultrasound, CT, or MRI.

Patients who underwent more frequent surveillance assessments were also classified as receiving structured surveillance. Patients who did not receive at least one annual surveillance examination (including clinical evaluation, tumor marker assessment, and imaging) were considered to have no structured surveillance.

### 2.2. Survival Definitions

Overall survival after surgery (OSaS) was defined as the time interval from surgery until death or last follow-up. Disease-free survival was defined as the time period from surgery until death or recurrence or last follow-up. In addition, overall survival after recurrence (OSaR) was defined as the time interval between diagnosis of recurrence and death or last follow-up. Time intervals are presented in months.

### 2.3. Surgical Procedures

All surgical resections were performed by experienced visceral surgeons specializing in pancreatic surgery. A standardized oncologic lymphadenectomy was performed in all PDAC resections. Depending on the tumor localization, pancreatic head resection, distal pancreatectomy, or total pancreatectomy was performed. Pancreatic head resection included an interaortocaval lymph node dissection, which led to some pM1 classifications when these lymph nodes were involved, as they are not considered locoregional pancreatic lymph nodes. When necessary, additional vascular or multivisceral resections were undertaken to achieve an R0 situation, with arterial vascular resections performed only in exceptional cases. No resection of the primary tumor was performed if liver metastases or peritoneal carcinomatosis were diagnosed intraoperatively.

### 2.4. Postoperative Course

Postoperatively, all cases were re-evaluated by our interdisciplinary tumor board considering all histopathological details. Adjuvant chemotherapy was generally recommended to all patients with an adequate general condition. The adjuvant chemotherapeutics included gemcitabine, gemcitabine + nab-paclitaxel, and FOLFIRINOX (5-fluorouracil, leucovorin, irinotecan, oxaliplatin) depending on the patient’s condition and the year. Some patients, despite having a good general condition, refused adjuvant chemotherapy.

Regular follow-up, including CT scans and CA19-9 level monitoring, was recommended to all patients. Additionally, patients were offered the option to undergo structured postoperative surveillance at the University Hospital Erlangen or to send external diagnostic results to our interdisciplinary tumor board.

### 2.5. Statistical Analysis

SPSS^®^ Version 28 (IBM, Armonk, NY, USA) was used to analyze the data. Data are presented as *n* (%) or median (interquartile range [IQR]). Comparisons of metric and ordinal data were calculated using the Kruskal–Wallis test. The Chi-square test was used for categorical data. A *p*-value ≤ 0.05 was considered statistically significant.

Survival curves were calculated using the Kaplan–Meier method and compared with the log-rank test. For survival analysis, the following variables were included in univariate and multivariate Cox regression models: age, neoadjuvant therapy, T category, N category, M category, tumor differentiation (G), adjuvant therapy, and structured surveillance as the primary endpoint of interest. These factors were selected based on their established clinical relevance and known prognostic significance. Tumor-related parameters (T, N, M, and G) reflect disease burden and biological aggressiveness [19]. Age- and treatment-related variables (neoadjuvant and adjuvant therapy) influence treatment eligibility and response and also reflect the patient’s general condition and tumor stage [21,22,23]. These factors were included as potential confounders to adjust the analysis accordingly, particularly since T and M categories, as well as neoadjuvant and adjuvant therapy, showed partially significant differences in baseline characteristics. Additional potentially prognostic variables—such as ASA score, comorbidities, and resection margin status (R status)—were not included in order to avoid overfitting of the multivariate model and to minimize potential collinearity, particularly between ASA/comorbidities and age.

## 3. Results

### 3.1. Dataset

Between 1 January 2005 and 31 December 2020, 295 patients underwent pancreatic resection due to PDAC at the University Hospital Erlangen. Among these, 248 patients (84%) had primary resection and 47 (16%) had resection following neoadjuvant (radio)chemotherapy. A total of 89 patients were excluded because of R2 status (*n* = 5), missing postoperative surveillance data (*n* = 41), or a DFS shorter than 90 days (*n* = 43). Therefore, 206 patients were included in our analyses. Of the 206 patients, 157 patients (76%) participated in a structured surveillance program after pancreatic resection, whereas 49 patients (24%) did not have structured surveillance (surveillance subgroups). During a mean follow-up of 28.5 months, 137 patients (67%) developed recurrence. After excluding 25 patients with missing data on the kind of recurrence (asymptomatic or symptomatic), the remaining 112 patients were grouped into those with recurrence diagnosed in scheduled surveillance examinations (46 patients; 41%) and those with recurrence diagnosed due to symptoms (66 patients; 59%) (recurrence subgroups) (Figure 1).

### 3.2. Patient Characteristics, Surgical Details, and Histopathological Results

There were no significant differences between the surveillance groups (with vs. without structured surveillance) regarding age, sex, ASA score, BMI, comorbidities, preoperative laboratory values, surgical details, R status, postoperative morbidity, or TNM category. However, the two surveillance groups differ significantly in terms of the prevalence of neoadjuvant therapy (27% vs. 10%, *p* = 0.018) and adjuvant chemotherapy (83% vs. 55%, *p* < 0.001) (Table 1, Table 2, Table 3 and Table 4).

Significant differences between the recurrence groups (recurrence in follow-up vs. symptomatic recurrence) regarding patient characteristics, surgical details, and histopathological results were found for cardiovascular comorbidity (4% vs. 20%, *p* = 0.023), T category (*p* = 0.045), and M category (0% vs. 12%, *p* = 0.020) (Table 1, Table 2, Table 3 and Table 4).

### 3.3. Follow-Up, Recurrence Details, and Overall and Disease-Free Survival

Recurrence occurred in 67% of our cohort, primarily as metastatic disease (85% of patients with recurrence). Isolated locoregional recurrence was detected significantly more often during follow-up compared to symptomatic detection (72% vs. 28%, *p* = 0.020) (Table 3 and Table 4).

**Figure 2:**
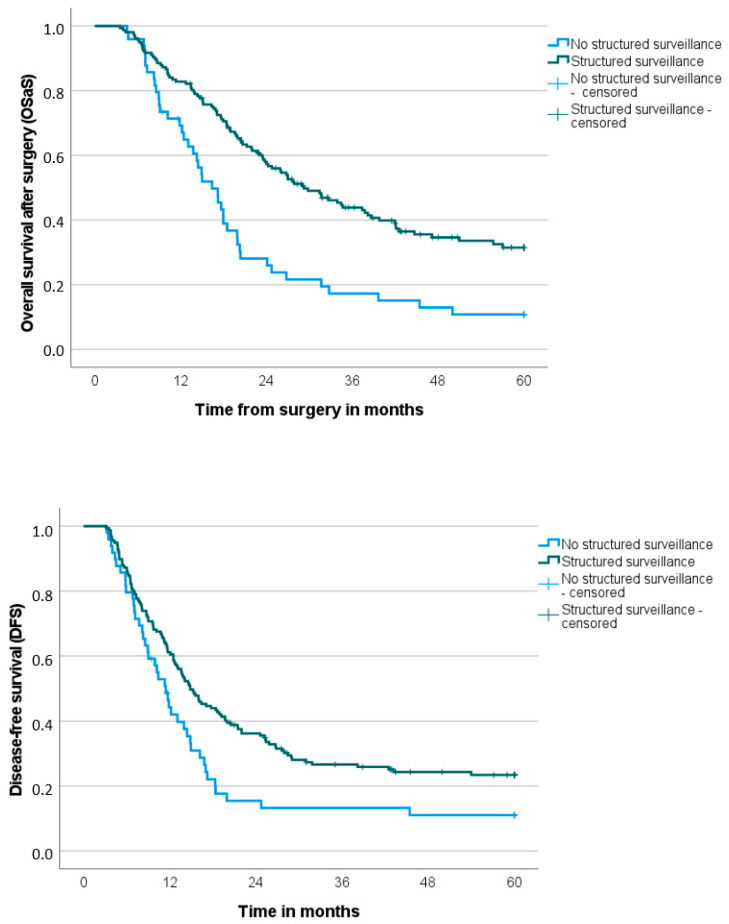
Overall survival after surgery (OSaS) and disease-free survival (DFS) stratified to performance of structured surveillance (yes vs. no) (*n* = 206).

We found significant survival advantages for both overall survival (OS) and disease-free survival (DFS) in patients with structured surveillance after PDAC resection. These patients exhibited longer overall survival from the time of surgery (OSaS), as well as longer overall survival after recurrence (OSaR) compared to patients without structured surveillance (OSaS: 29.2 months with structured surveillance vs. 16.4 months without, *p* < 0.001; OSaR: 10.8 months vs. 3.6 months, *p* < 0.001). The difference in disease-free survival was also significant (14.8 months with structured surveillance vs. 11.4 months without, *p* < 0.010) (Table 3 and Figure 2). 

**Figure 3:**
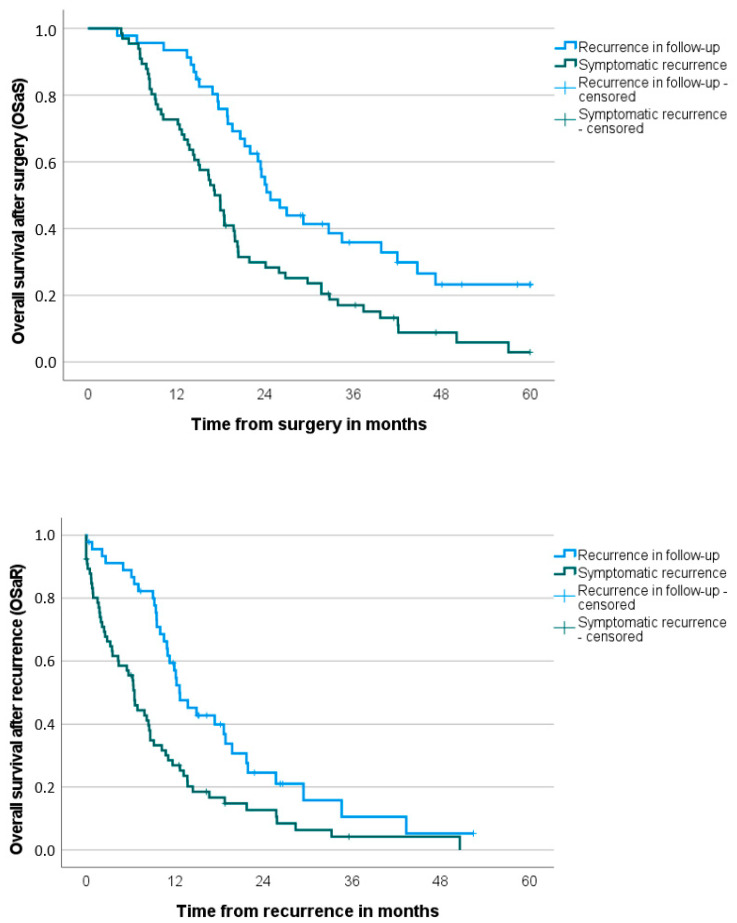
Overall survival after surgery (OSaS) and overall survival after recurrence (OSaR) stratified to kind of recurrence (in follow-up vs. symptomatic) (*n* = 112).

Significant differences in OS were observed between the recurrence in follow-up group and the symptomatic recurrence group, with the recurrence in follow-up group showing longer survival rates. The OSaS in the recurrence in follow-up group was 24.8 months versus 17.2 months in the symptomatic recurrence group (*p* < 0.001). The OSaR was also longer in the recurrence in follow-up group (12.6 months vs. 6.5 months in the symptomatic recurrence group, *p* < 0.001) (Table 4 and Figure 3).

### 3.4. Prognostic Factors for Overall Survival After Surgery (OSaS) and Disease-Free Survival (DFS) in the Whole Patient Cohort (n = 206)

Selected potentially prognostic factors regarding OSaS and DFS after PDAC resection are shown in Table 5. Missing structured surveillance was identified as an independent negative prognostic factor for both OS (OR 1.8, 95% CI 1.2–2.9, *p* = 0.006) and DFS (OR 1.4, 95% CI 1.0–2.2, *p* = 0.048). Multivariate analysis also revealed that age > 70 years (OR 1.6, 95% CI 1.1–2.3, *p* = 0.022), neoadjuvant therapy (OR 0.4, 95% CI 0.2–0.7, *p* = 0.001), lymph node metastases (OR 2.2, 95% CI 1.4–3.4, *p* < 0.001), distant metastases (OR 2.7, 95% CI 1.3–5.7, *p* = 0.009), poor differentiation (grade 3) (OR 1.7, 95% CI 1.1–2.5, *p* = 0.012), and missing adjuvant chemotherapy (OR 1.8, 95% CI 1.1–2.9, *p* = 0.017) were independent negative prognostic factors for OS. For DFS, lymph node metastasis (OR 1.9, 95% CI 1.3–2.9, *p* = 0.001) was identified as an additional independent negative prognostic factor (Table 5).

### 3.5. Prognostic Factors for Overall Survival After Surgery (OSaS) and Overall Survival After Recurrence (OSaR) in Patients with Recurrence (n = 112)

The kind of recurrence (follow-up vs. symptomatic) was identified as an independent prognostic factor for OSaS and OSaR (OSaS: OR 2.2, 95% CI 1.3–3.7, *p* = 0.003; OSaR: OR 1.9, 95% CI 1.2–3.2, *p* = 0.007). For OSaS, neoadjuvant therapy (OR 0.3, 95% CI 0.1–0.5, *p* < 0.001) and lymph node metastases (OR 2.2, 95% CI 1.3–3.7, *p* = 0.002) were additional independent prognostic factors in the multivariate analysis. For OSaR, age > 70 years, neoadjuvant therapy, lymph node and distant metastases, G3 differentiation, and missing adjuvant chemotherapy were found to be additional independent prognostic factors (age: OR 1.6, 95% CI 1.0–2.6, *p* = 0.038; neoadjuvant therapy: OR 0.3, 95% CI 0.1–0.6, *p* < 0.001; N status: OR 1.8, 95% CI 1.1–3.0, *p* = 0.030; M status: OR 5.9, 95% CI 2.2–15.6, *p* < 0.001; differentiation: OR 2.0, 95% CI 1.2–3.3, *p* = 0.008; and adjuvant chemotherapy: OR 2.0, 95% CI 1.1–3.6, *p* = 0.024) (Table 6).

## 4. Discussion

This study analyzed the impact of a structured surveillance after PDAC resection on overall and disease-free survival, given a wide variety of recommendations and clinical practice in the different countries and societies without high-level evidence [11,12,13,14,15]. It provides valuable clinical data from a German Pancreatic Cancer Center not only for the benefit of structured surveillance after pancreatic cancer resection, but also for the advantage of detection of asymptomatic cancer recurrence. A recent systematic review by Daamen et al. found conflicting results for surveillance after PDAC resection and emphasized the lack of evidence [18]. Similar findings are reported by Ansari et al. in a recent review, also containing a survey of the surveillance practice in Nordic European countries. The authors state that although postoperative surveillance is regularly provided in most of the investigated centers, the protocols differ [24].

Despite previously contradictory findings, we found a clear advantage with a considerably longer overall survival (OS) and disease-free survival (DFS) for patients with structured surveillance after PDAC resection compared to those without surveillance (OS after surgery [OSaR: 29.2 months with surveillance vs. 16.4 months without surveillance], OS after recurrence [OSaR: 10.8 months vs. 3.6 months], and DFS [14.8 months vs. 11.4 months]). Considering only those patients suffering from recurrence, an OS advantage was observed again for those with recurrence detected in follow-up (OSaS: 24.8 months, OSaR: 12.6 months) compared to those with symptomatic recurrence (OSaS: 17.2 months, OSaR: 6.5 months).

When considering our data and results, it is important to acknowledge the potential bias arising from differences in the comparison cohorts, particularly the higher rate of neoadjuvant and adjuvant chemotherapy in the structured surveillance group and the worse T and M category in the group of patients with symptomatic recurrence. However, all these parameters were included in the multivariate survival analysis, and the survival advantages regarding overall survival and disease-free survival were independently confirmed for both the structured surveillance group and the group with recurrence detected during follow-up.

Our results are supported by further evidence suggesting that a structured surveillance program may improve overall and disease-free survival after PDAC resection by allowing earlier detection of recurrence. Similar to our findings, Zhang et al. reported a favorable OS for patients with asymptomatic recurrence detected during scheduled follow-up compared to those patients with symptomatic recurrence (24.8 months vs. 15.1 months) [17]. In a similar retrospective study, Gonzales et al. also found a better outcome for patients with asymptomatic recurrence [25]. Halle-Smith et al. stated in a review and meta-analysis that structured surveillance results in more patients being diagnosed with asymptomatic recurrence, leading to better outcomes [16]. Tjaden et al. emphasized the importance of structured follow-up, including imaging, to detect cancer recurrence early, particularly in asymptomatic patients, allowing for timely therapeutic interventions. They also highlighted the benefits of follow-up in specialized care centers to optimize symptom-directed medical treatment after pancreatic resection [26]. Luu et al. found evidence for structured follow-up programs for patients with long-term survival after pancreatic resection, as those patients typically present with asymptomatic cancer recurrence [27]. In a recently published prospective trial, Andel et al. reported that patients with a structured follow-up with routine imaging after pancreatic resection were more likely to receive recurrence-focused treatment and showed a favorable OS [28].

However, there are also some studies showing no survival benefit from structured surveillance after PDAC resection. Tzeng et al. evaluated the cost-effectiveness of different structured surveillance modalities and found only increasing cost without a survival benefit for intensified surveillance including imaging [29]. Likewise, Witkowski et al. reported an increasing number of CT scans without survival benefit for patients with regular imaging after pancreatic resection [30]. However, these studies recruited patients from the 1990s until 2008 or 2005, respectively, when less effective chemotherapy regimens were available compared to today, potentially reducing the positive prognostic effect of early recurrence detection [5]. Moreover, Nong et al. reported recent evidence for CA 19-9 as a valid marker to identify recurrence [31], which may be a further stratification tool to avoid cost-intensive imaging for patients with low recurrence risk.

Some other considerations regarding the impact of structured surveillance after PDAC resection should be addressed.

First, there is no internationally approved definition for structured surveillance, as follow-up examinations may include clinical examinations, CA 19-9 levels, ultrasound, MRI, or CT scans. Consequently, it may be difficult to compare the results of different studies concerning this topic. In this study, the focus is on clinical examination and imaging, and we defined “structured surveillance” as at least one clinical and imaging examination per year. This means there has to be an examination on a regular, at least yearly, basis, although the applied schedules may differ. Thus, from our results, it is not possible to state if the frequency or the chosen imaging has an influence. However, the effect of examinations every three or six months may even be bigger.

**Figure 4:**
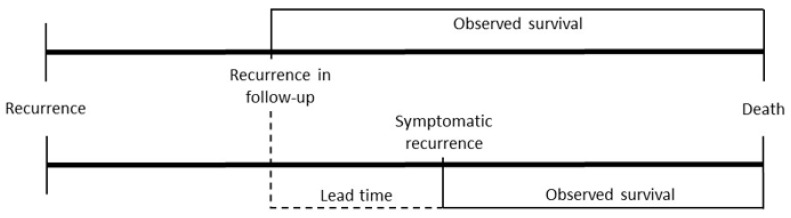
Lead time bias: overestimation of survival because of earlier recurrence diagnosis in follow-up.

Secondly, concerning early detection of recurrence, it is essential to discuss lead time and length time biases, both of which can inflate the perceived benefits of structured surveillance [32,33]. Lead time bias refers to the overestimation of survival because patients diagnosed through screening or follow-up appear to survive longer, even though the time of diagnosis does not affect the actual time of death (Figure 4). Length time bias reflects that slower-growing tumors provide a longer detection window during follow-up compared to aggressive tumors [32]. To mitigate these biases, we analyzed not only overall survival (OS) after recurrence, but also OS after surgery, consistently finding significantly longer OS associated with structured surveillance, including among those experiencing recurrence. Furthermore, we found a significant difference in not only OS, but also DFS, possibly meaning that the influence of the lead time bias is negligible in our cohort, although this fact might also point to a relevant selection bias or a length time bias, as a slow-growing tumor might be detectable later and, therefore, the disease-free period seems to be longer.

Thirdly, the psychological impact of structured surveillance programs on patients is crucial, with many reporting anxiety and fear of cancer recurrence due to routine follow-up exams [34]. Despite these concerns, patients also express a need for reassurance provided by surveillance following pancreatic cancer resection [35]. Effective doctor–patient communication is vital in addressing these emotional aspects within surveillance programs.

Fourth, in addition to structured follow-up aimed at improving survival, follow-up may be essential for quality of life. Aspects such as nutrition, postoperative monitoring, and further supportive measures can be crucial for improving quality of life. The aspect of quality of life could not be addressed in our study due to its retrospective design.

Finally, the financial implications for health care systems due to extensive follow-up examinations should not be overlooked [29].

The present study has several limitations: Although the single center study design ensures a homogeneous therapy approach, generalizing the results may be challenging. Furthermore, as it is a retrospective analysis of prospectively collected data, there might be some bias. Third, the number of patients is limited, especially regarding the long observation period of 15 years. This duration spans a period when chemotherapy regimens evolved, potentially introducing bias into the analysis. Fourth, multiple factors influence the decision of whether a patient undergoes follow-up, many of which are likely not fully reflected by the parameters we analyzed. Fifth, we collected data about surveillance and recurrence not only in our clinical information systems and cancer registry, but also by contacting patients, their families, and general practitioners. This may lead to deficiencies in the collected data. To avoid inaccuracies and inaccurate data collection, we included only medical reports in our data collection. Sixth, our study did not further investigate the treatment of recurrence, which also plays a crucial role in determining the value of structured surveillance. Only patients for whom a viable treatment option is available can truly benefit from early detection of recurrence, and asymptomatic patients might have a better performance status and, therefore, be more likely to receive therapy. Seventh, the subgroup analyses, particularly those based on recurrence cases (*n* = 112), may be limited by reduced statistical power, increasing the risk of Type I and Type II errors.

## 5. Conclusions

The present study provides additional evidence supporting prolonged overall and disease-free survival in patients undergoing structured surveillance following resection of pancreatic cancer. However, prospective randomized clinical trials are needed to establish high-level evidence and validate these findings.

## Figures and Tables

**Table 1:** Characteristics of patients undergoing curative pancreatic resection for pancreatic ductal adenocarcinoma stratified to postoperative structured surveillance (yes vs. no) (*n* = 206).

	All Patients (*n* = 206)		
	Structured Surveillance	No Structured Surveillance	*p*-Value
**Number**	157	49	
**Age (years), median (IQR)**	67 (14)	70 (11)	0.051
**Sex, *n* (%)**			0.513
** Female**	77 (49)	21 (43)	
** Male**	80 (51)	28 (57)	
ASA (*n* = 202/110) *, *n* (%)			0.198
** I**	4 (3)	0 (0)	
** II**	97 (64)	29 (59)	
** III**	52 (34)	20 (41)	
**BMI (kg/m^2^), median (IQR)**	25.4 (5.0)	25.4 (5.4)	0.387
**Comorbidity, *n* (%)**			
** Hypertension**	83 (53)	22 (45)	0.413
** Diabetes**	39 (25)	15 (31)	0.459
** Cardiovascular**	22 (14)	13 (27)	0.051
** Pulmonary**	10 (6)	3 (6)	1.000
** Cerebrovascular**	6 (4)	2 (4)	1.000
** Liver disease**	16 (10)	5 (10)	0.281
**Neoadjuvant therapy, *n* (%)**	42 (27)	5 (10)	**0.018**
**Preoperative albumin (g/L), median (IQR)**	40.2 (7.5)	38.8 (6.4)	0.623
Preoperative CA19-9 (U/mL) (*n* = 135) *, median (IQR)	90 (326)	64 (227)	0.484
**Preoperative CEA (ng/mL), median (IQR)**	2.3 (3.0)	2.2 (2.7)	0.986

ASA = American Society of Anesthesiologists classification; BMI = body mass index. * Missing data.

**Table 2:** Characteristics of patients undergoing curative pancreatic resection for pancreatic ductal adenocarcinoma stratified to kind of recurrence (in follow-up vs. symptomatic) (*n* = 112).

	Only Patients with Recurrence (*n* = 112) *		
	Recurrence in Follow-Up	Symptomatic Recurrence	*p*-Value
**Number**	46	66	
**Age (years), median (IQR)**	64 (19)	69 (13)	0.069
**Sex, *n* (%)**			0.081
** Female**	24 (52)	23 (35)	
** Male**	22 (48)	43 (65)	
ASA (*n* = 202/110) **, *n* (%)			0.904
** I**	0 (0)	1 (1)	
** II**	30 (67)	44 (68)	
** III**	15 (33)	20 (31)	
**BMI (kg/m^2^), median (IQR)**	26.3 (6.9)	25.6 (5.0)	0.280
**Comorbidity, *n* (%)**			
** Hypertension**	21 (46)	32 (49)	0.848
** Diabetes**	7 (15)	20 (30)	0.076
** Cardiovascular**	2 (4)	13 (20)	**0.023**
** Pulmonary**	3 (7)	4 (6)	1.000
** Cerebrovascular**	1 (2)	2 (3)	1.000
** Liver disease**	5 (11)	5 (8)	0.846
**Neoadjuvant therapy, *n* (%)**	14 (30)	14 (21)	0.375
**Preoperative albumin (g/L), median (IQR)**	41.1 (6.2)	39.1 (6.9)	0.188
Preoperative CA19-9 (U/mL) (*n* = 135) **, median (IQR)	72 (443)	133 (489)	0.775
**Preoperative CEA (ng/mL), median (IQR)**	2.9 (3.5)	2.3 (2.7)	0.485

ASA = American Society of Anesthesiologists classification; BMI = body mass index. * Data about kind of recurrence only available in 112 patients. ** Missing data.

**Table 3:** Surgical and histopathological details of patients undergoing curative pancreatic resection for pancreatic ductal adenocarcinoma stratified to postoperative structured surveillance (yes vs. no) (*n* = 206).

	All Patients (*n* = 206)		
	Structured Surveillance (*n* = 157)	No Structured Surveillance (*n* = 49)	*p*-Value
**Kind of surgery, *n* (%)**			0.874
** Pancreatic head resection**	119 (76)	39 (80)	
** Distal pancreatectomy**	34 (22)	9 (18)	
** Total pancreatectomy**	4 (3)	1 (2)	
**Vascular resection, *n* (%)**	56 (36)	15 (31)	0.607
**Multivisceral resection, *n* (%)**	40 (26)	13 (27)	1.000
**Postoperative In-hospital morbidity, *n* (%)**	75 (48)	28 (57)	0.326
**Postoperative in-hospital major morbidity, *n* (%)**	43 (27)	10 (20)	0.357
T category (*n* = 205/112) *, *n* (%)			0.193
** (y)pT0**	3 (1)	1 (2)	
** (y)pT1**	14 (9)	9 (18)	
** (y)pT2**	48 (31)	10 (20)	
** (y)pT3**	89 (57)	29 (59)	
** (y)pT4**	3 (2)	0 (0)	
**N category, *n* (%)**			0.518
** (y)pN0**	79 (50)	22 (45)	
** (y)pN+**	78 (50)	27 (55)	
**M category, *n* (%)**			0.485
** M0**	149 (95)	45 (92)	
** (y)pM1**	8 (5)	4 (8)	
**R status, *n* (%)**			0.301
** R0**	146 (93)	48 (98)	
** R1**	11 (7)	1 (2)	
Differentiation (*n* = 182/96) *, *n* (%)			0.859
** G1/2**	54 (39)	16 (36)	
** G3**	84 (61)	28 (64)	
**Adjuvant chemotherapy, *n* (%)**	127 (83)	23 (55)	**<0.001**
**Recurrence, *n* (%)**	105 (67)	32 (65)	0.863
Location of recurrence (*n* = 130/106) *, *n* (%)			0.140
** Locoregional only**	19 (18)	1 (4)	
** Metastatic disease**	56 (54)	19 (70)	
** Both**	28 (27)	7 (26)	
**Kind of recurrence (*n* = 112), *n* (%)**			**<0.001**
** In follow-up**	46 (57)	0 (0)	
** Symptomatic**	35 (43)	31 (100)	
**Time to recurrence (months), median (SD)**	12.1 (11.4)	9.9 (8.3)	**0.029**
**Overall survival from surgery (months), median (SD)**	29.2 (3.2)	16.4 (1.8)	**<0.001**
**Overall survival from recurrence (months) (*n* = 137/112), median (SD)**	10.8 (1.0)	3.6 (2.1)	**<0.001**
**Disease-free survival (months), median (SD)**	14.8 (1.3)	11.4 (1.1)	**0.010**

SD = standard deviation. * Missing data.

**Table 4:** Surgical and histopathological details of patients undergoing curative pancreatic resection for pancreatic ductal adenocarcinoma stratified to kind of recurrence (in follow-up vs. symptomatic) (*n* = 112).

	Only Patients with Recurrence (*n* = 112) *		
	Recurrence in Follow-Up (*n* = 46)	Symptomatic Recurrence (*n* = 66)	*p*-Value
**Kind of surgery, *n* (%)**			1.000
** Pancreatic head resection**	35 (76)	51 (77)	
** Distal pancreatectomy**	11 (24)	15 (23)	
** Total pancreatectomy**	0 (0)	0 (0)	
**Vascular resection, *n* (%)**	14 (30)	25 (38)	0.430
**Multivisceral resection, *n* (%)**	14 (30)	15 (23)	0.387
**Postoperative In-hospital morbidity, *n* (%)**	21 (46)	31 (47)	1.000
**Postoperative in-hospital major morbidity, *n* (%)**	15 (33)	12 (18)	0.063
T category (*n* = 205/112) **, *n* (%)			**0.045**
** (y)pT0**	0 (0)	2 (3)	
** (y)pT1**	8 (17)	7 (11)	
** (y)pT2**	12 (26)	12 (18)	
** (y)pT3**	23 (50)	45 (68)	
** (y)pT4**	3 (7)	0 (0)	
**N category, *n* (%)**			0.699
** (y)pN0**	22 (48)	28 (42)	
** (y)pN+**	24 (52)	38 (58)	
**M category, *n* (%)**			**0.020**
** M0**	46 (100)	58 (88)	
** (y)pM1**	0 (0)	8 (12)	
**R status, *n* (%)**			1.000
** R0**	43 (94)	63 (94)	
** R1**	3 (6)	4 (6)	
Differentiation (*n* = 182/96) **, *n* (%)			0.058
** G1/2**	20 (49)	16 (29)	
** G3**	21 (51)	39 (71)	
**Adjuvant chemotherapy, *n* (%)**	40 (87)	47 (72)	0.100
**Recurrence, *n* (%)**	46 (100)	66 (100)	-
Location of recurrence (*n* = 130/106) **, *n* (%)			**0.020**
** Locoregional only**	13 (28)	5 (8)	
** Metastatic disease**	23 (50)	35 (58)	
** Both**	10 (22)	20 (33)	
**Kind of recurrence (*n* = 112), *n* (%)**			**-**
** In follow-up**	46 (100)	0 (0)	
** Symptomatic**	0 (0)	66 (100)	
**Time to recurrence (months), median (SD)**	12.6 (12.3)	10.1 (8.6)	0.054
**Overall survival from surgery (months), median (SD)**	24.8 (2.2)	17.2 (1.1)	**<0.001**
**Overall survival from recurrence (months) (*n* = 137/112), median (SD)**	12.6 (1.6)	6.5 (0.7)	**<0.001**
**Disease-free survival (months), median (SD)**	-	-	-

SD = standard deviation. * Data about kind of recurrence only available in 112 patients. ** Missing data.

**Table 5:** Selected prognostic factors for overall survival after surgery (OSaS) and disease-free survival (DFS) in all patients with resected pancreatic ductal adenocarcinoma (*n* = 206).

		Overall Survival After Surgery (OSaS)					Disease-Free Survival (DFS)				
		Univariate		Multivariate			Univariate		Multivariate		
	*n*	Median OS	*p*	OR	95% CI	*p*	Median DFS	*p*	OR	95% CI	*p*
**Age**			**0.007**	**1.6**	**1.1–2.3**	**0.022**		0.140	1.3	0.9–1.9	0.138
** ≤70 years**	127	29.0					13.8				
** >70 years**	79	20.3					14.6				
**Neoadjuvant therapy**			0.623	**0.4**	**0.2–0.7**	**0.001**		0.500	0.6	0.3–1.1	0.083
** Yes**	47	26.0					11.0				
** No**	159	24.1					14.0				
T category (*n* = 205) *			0.144	1.0	0.7–1.6	0.889		0.313	1.0	0.7–1.4	0.853
** (y)pT0/(y)pT1/(y)pT2**	84	31.7					16.0				
** (y)pT3/(y)pT4**	121	22.0					13.1				
**N category**			**<0.001**	**2.2**	**1.4–3.4**	**<0.001**		**0.001**	**1.9**	**1.3–2.9**	**0.001**
** (y)pN0**	101	33.9					**17.1**				
** (y)pN+**	105	19.9					**11.9**				
**M category**			**<0.001**	**2.7**	**1.3–5.7**	**0.009**		**0.042**	1.5	0.7–3.0	0.307
** M0**	194	26.0					**14.4**				
** (y)pM1**	12	10.2					**9.0**				
Differentiation (*n* = 182) *			**0.039**	**1.7**	**1.1–2.5**	**0.012**		0.087	1.4	1.0–2.0	0.087
** G1/G2**	70	31.7					18.4				
** G3**	112	20.3					13.6				
**Adjuvant chemotherapy**			0.195	**1.8**	**1.1–2.9**	**0.017**		0.602	1.3	0.9–2.1	0.184
** Yes**	150	25.9					14.0				
** No**	45	18.5					13.1				
**Structured surveillance**			**<0.001**	**1.8**	**1.2–2.9**	**0.006**		**0.010**	**1.4**	**1.0–2.2**	**0.048**
** Yes**	157	29.2					**14.8**				
** No**	49	16.4					**11.4**				

ASA = American Society of Anesthesiologists classification. * Missing data.

**Table 6:** Selected prognostic factors for overall survival after surgery (OSaS), overall survival after recurrence (OSaR), and disease-free survival (DFS) in patients with resected pancreatic ductal adenocarcinoma and known kind of recurrence (*n* = 112).

		Overall Survival After Surgery (OSaS)					Overall Survival After Recurrence (OSaR)				
		Univariate		Multivariate			Univariate		Multivariate		
	*n*	Median OS	*p*	OR	95% CI	*p*	Median OS	*p*	OR	95% CI	*p*
**Age**			0.160	1.4	0.9–2.3	0.139		**0.024**	**1.6**	**1.0–2.6**	**0.038**
** ≤70 years**	69	23.4					10.7				
** >70 years**	43	18.5					7.9				
**Neoadjuvant therapy**			0.330	**0.3**	**0.1–0.5**	**<0.001**		0.844	**0.3**	**0.1–0.6**	**<0.001**
** Yes**	28	18.9					10.0				
** No**	84	20.3					9.2				
**T category**			0.133	0.9	0.5–1.7	0.932		**0.014**	1.3	0.8–2.3	0.296
** (y)pT0/(y)pT1/(y)pT2**	41	23.4					12.7				
** (y)pT3/(y)pT4**	71	19.9					8.4				
**N category**			**0.013**	**2.2**	**1.3–3.7**	**0.002**		**0.022**	**1.8**	**1.1–3.0**	**0.030**
** (y)pN0**	50	27.0					12.2				
** (y)pN+**	62	18.0					7.1				
**M category**			**<0.001**	2.5	1.0–6.4	0.052		**<0.001**	**5.9**	**2.2–15.6**	**<0.001**
** M0**	104	20.4					12.2				
** (y)pM1**	8	9.9					0.9				
Differentiation (*n* = 96) *			0.088	1.5	0.9–2.6	0.105		**0.034**	**2.0**	**1.2–3.3**	**0.008**
** G1/G2**	36	25.9					12.1				
** G3**	60	18.5					7.9				
**Adjuv. chemoth.**			0.280	2.2	1.2–4.1	0.013		0.186	**2.0**	**1.1–3.6**	**0.024**
** Yes**	87	20.7					10.2				
** No**	24	15.0					6.3				
**Kind of recurrence**			**<0.001**	**2.2**	**1.3–3.7**	**0.003**		**<0.001**	**1.9**	**1.2–3.2**	**0.007**
** In follow-up**	46	24.8					12.6				
** Symptomatic**	66	17.2					6.5				

ASA = American Society of Anesthesiologists classification. * Missing data.

## Data Availability

All data generated or analyzed during this study are included in this published article.
